# Multi-Factor Clustering Incorporating Cell Motility Predicts T Cell Expansion Potential

**DOI:** 10.3389/fcell.2021.648925

**Published:** 2021-04-09

**Authors:** Joanne H. Lee, Shuai Shao, Michelle Kim, Stacey M. Fernandes, Jennifer R. Brown, Lance C. Kam

**Affiliations:** ^1^Department of Biomedical Engineering, Columbia University, New York, NY, United States; ^2^Department of Medical Oncology, Harvard Medical School, Dana-Farber Cancer Institute, Boston, MA, United States

**Keywords:** T cell, Leukemia, machine learning, immunotherapy, cell migration

## Abstract

Expansion of an initial population of T cells is essential for cellular immunotherapy. In Chronic Lymphocytic Leukemia (CLL), expansion is often complicated by lack of T cell proliferation, as these cells frequently show signs of exhaustion. This report seeks to identify specific biomarkers or measures of cell function that capture the proliferative potential of a starting population of cells. Mixed CD4+/CD8+ T cells from healthy donors and individuals previously treated for CLL were characterized on the basis of proliferative potential and *in vitro* cellular functions. Single-factor analysis found little correlation between the number of populations doublings reached during expansion and either Rai stage (a clinical measure of CLL spread) or PD-1 expression. However, inclusion of *in vitro* IL-2 secretion and the propensity of cells to align onto micropatterned features of activating proteins as factors identified three distinct groups of donors. Notably, these group assignments provided an elegant separation of donors with regards to proliferative potential. Furthermore, these groups exhibited different motility characteristics, suggesting a mechanism that underlies changes in proliferative potential. This study describes a new set of functional readouts that augment surface marker panels to better predict expansion outcomes and clinical prognosis.

## Introduction

T cells have emerged as a compelling agent in the treatment of diseases ranging from cancer to autoimmunity. However, clinical use of T cells as a therapy relies on the production of cells of sufficient quantity and quality from a small starting population; the inability of an individual’s cells to carry out this expansion would make a cellular approach inappropriate for both therapy and participation in clinical trials (Frey, 2015). This poses a particular challenge as disease state often dampens immune function and response including expansion. As a key example, T cells from individuals with Chronic Lymphocytic Leukemia (CLL) show defects in expansion and subsequent function (Wherry, 2011; Tonino et al., 2012; Riches et al., 2013; Palma et al., 2017; McLane et al., 2019), which resembles exhaustion and is associated with lower remission of CLL than Acute Lymphoblastic Leukemia through autologous CAR-T cell therapy (Maude et al., 2014; Porter et al., 2015). CLL is also associated with higher levels of key exhaustion markers such as PD-1, TIM-3, LAG-3, CTLA-4, TIGIT, and CD160 (Wherry, 2011; Long et al., 2015; McClanahan et al., 2015; Wherry and Kurachi, 2015), as well as deficits in cell function such as migration and formation of immune synapse structures (Ramsay et al., 2008, 2012, 2013). However, a clear understanding of how biomarkers are associated with cellular function, disease progression, and potential treatment remains elusive. Using a machine learning approach, this report seeks to develop a framework for combining molecular biomarkers, measures of cell function, and other inputs to characterize T cells from individuals with CLL, ultimately in an effort to improve production of cells for cellular immunotherapy.

## Materials and Methods

### Cell Culture

Mixed CD4^+^/CD8^+^ populations of primary human T cells were isolated from peripheral blood lymphocyte fractions (Leukopaks, New York Blood Center) by negative selection (Rosette-Sep kit, Stem Cell Technology) and density centrifugation (Ficoll-Paque PLUS, GE). Mixed CD4^+^/CD8^+^ T cells from individuals who were previously treated for CLL were purified using identical selection techniques. Clinical biomarkers were collected over the course of treatment. In particular, Rai stage, a standardized measure of CLL spread, was determined during patient care from blood tests (cell counts) and physical exams (tissue enlargement). For all experiments, cells were cultured in RPMI 1640 supplemented with 10% fetal bovine serum, 10 mM HEPES, 2 mM L-glutamine, 50 U/mL penicillin, 50 μg/mL streptomycin, and 50 μM β-mercaptoethanol (Sigma or Life Technologies, unless otherwise noted). T cell populations were analyzed for PD-1 expression by flow cytometry using α-PD-1 (PE-Cy7, clone EH12.2H7, Biolegend).

### Design and Fabrication of Microscopy Chambers

Conical-well, open-bottom wells were used to improve the efficiency of microscopy-based cell function analysis. Individual wells had a cylindrical well geometry of 5 mm in internal diameter and 4.5 mm depth, but with a 45° conical bottom ending with a 1-mm diameter opening at the bottom of the structure. Multiple wells in a 2 × 4 rectangular array were arranged into chambers following the layout and center-to-center distance of standard 96-well plates. Chambers were fabricated out of polypropylene by injection molding (Protolabs). For use in microscopy, chambers were affixed onto test surfaces using transfer tape (3 M) that was laser cut to provide correct overall dimensions and provide holes for the 1-mm openings.

### Surface Micropatterning

Micropatterned surfaces were created by microcontact printing (20, 21). Briefly, glass coverslips were patterned with 2-μm diameter circular features of activating proteins, spaced in square arrays at a center-to-center distance of 15 μm. Microcontact printing was carried out by coating topographically defined, polydimethylsiloxane stamps with a mixture of α-CD3 (clone OKT3, Bio X Cell) and α-CD28 (clone 9.3, Bio X Cell) antibodies. The strength of TCR/CD3 activating signal was modulated by changing the amount of α-CD3 in the stamping solutions, which contained α-CD28 at 15 μg/ml, α-CD3 at a specified concentration (5, 3, 1.5, or 1 μg/ml), and an inert antibody (chicken α-goat IgG, Life Technologies) for a total concentration to 20 μg/ml. The strength of α-CD3 signal was expressed as percent of antibody solution associated with OKT3 (e.g., 15 μg/ml α-CD28 + 3 μg/ml α-CD3+ 2 μg/ml α-gt was denoted as 15% OKT3). A microscopy chamber was then adhered onto the coverslips, aligning the wells with the patterned regions. Finally, open areas of the coverslip were coated with 2 μg/ml of ICAM-1 (ICAM-1/Fc chimera protein, R&D Systems).

### Expansion

Assays of cell expansion were carried out as previously described (O’Connor et al., 2012; Dang et al., 2018). Briefly, mixed CD4^+^/CD8^+^ populations of 1 × 10^6^ T cells were stimulated with Human T-Activator CD3/CD28 Dynabeads (ThermoFisher) at a bead to cell ratio of 3:1 on day 0 of an expansion process. On day 3 and every second day after that, the number of T cells was counted, and additional media added to reduce cell concentration to 5 × 10^5^ cells/ml. Proliferative capacity was quantified as the maximum number of doublings achieved over the expansion, after which cell number decreased; the expansion process was terminated at that point.

### Microscopy-Based Assays of Cell Function

Cell alignment, motility, and IL-2 secretion assays were carried out by seeding 1 × 10^4^ T cells in a 50 μl volume into prepared microscopy chambers attached to micropatterned coverslips or other experimental surface. Cell culture was carried out under standard conditions (37°C, humidified environment, 5% CO_2_ environment).

Cell alignment and IL-2 secretion were measured 6 h after seeding. IL-2 secretion was measured using a surface capture method (Shen et al., 2008; Bashour et al., 2014). Briefly, cells were incubated with a bi-reactive antibody, which binds to the T cell surface and presents a site for IL-2 capture. Secreted IL-2 is captured over the course of the 6 h incubation, and then detected using an APC-labeled α-IL2 antibody. Cells were fixed with 4% paraformaldehyde. Amplification of the IL-2 signal was provided by incubation with a tertiary, biotinylated α-APC antibody followed by streptavidin-AF647. Interference reflection microscopy provided an outline of each cell, which was used to determine the fraction of cells that had aligned with an activating pattern. Fluorescence imaging allowed cell-by-cell measurement of surface-captured IL-2, which was collected for cells aligned to the patterns.

Cell motion was recorded by live-cell microscopy in the first hour after seeding using a stage top incubator (Tokai). Images were collected at 30 s intervals over the 60 min observation period. Only T cells with fully formed lamellipodia were considered for motility analysis. Velocity was defined as average velocity before cells stopped on an activation feature. A stop was defined as a cessation of overall motion for longer than 3 min, thus not including encounters where cells crossed a feature without halting. For a subset of experiments, T cells were stained with α-PD1 BB515 (clone EH12.1, Becton Dickinson) prior to seeding.

### Statistics and Analysis

Analysis of donor cells was carried out in the R and MATLAB software environments. To identify the smallest set of factors that can account for the majority of variance in the donor data set (Supplementary Table 1), Factor Analysis of Mixed Data (FAMD) was carried out using the “FactoMineR” and “factoextra” libraries in R. Sex and IgVH were treated as categorical factors. Rai stage, represented by the integer associated with the analysis (0–4) was rank transformed and then treated as a numerical factor, noting that increasing Rai stage corresponds to greater CLL spread. Missing data was imputed by Multiple Imputation by Chained Equations (MICE) using the “mice” library in R. Numerical data was normalized (mean = 0, standard deviation = 1) prior to analysis by FAMD. Once variables to be included for clustering were identified, data was analyzed by k-medoids using the “cluster” library in R. Resampling analysis was carried out using the R “boot” library. MATLAB was used to reconcile cluster assignments between runs.

Quantitative comparisons between multiple conditions were carried out using two-tailed ANOVA methods. When validated by ANOVA (α = 0.05), comparison of data between multiple conditions was carried out using Tukey’s honest significance test methods. As specified in the figure captions, data were alternatively analyzed using Kruskal-Wallis test by ranks (α = 0.05). These tests, including permutation analysis when specified, were carried out using the MATLAB software environment.

### Study Approval

All experiments were performed in accordance with protocols approved by either the Dana-Farber Cancer Institute or Columbia University. Clinical information was provided from patient records from the Dana-Farber Cancer Institute. Informed consent was obtained for each patient on an ongoing research protocol approved by the Dana-Farber Cancer Institute Institutional Review Board (no. 99-224).

## Results

### CLL T Cells Show Reduced Proliferative Capacity

**FIGURE 1:**
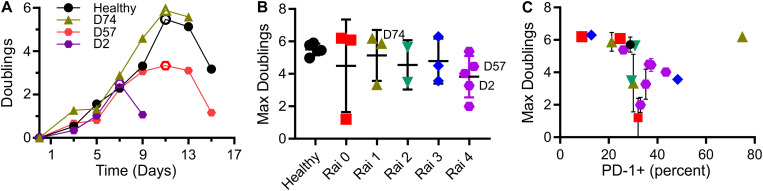
T cells from CLL patients show deficits in expansion. **(A)** Timecourse of expansion for cells from three individuals, including a control condition of cells from a healthy donor, a CLL patient with cells showing moderate deficit in expansion (D57), and one with minimal proliferative potential (D2). The maximum number of doublings reached over an experiment (indicated by the open symbols) was used as a single, characteristic measure of expansion. **(B)** Maximum doublings for cells as a function of Rai stage (not including healthy donors) were compared by permutation analysis applied to Kruskal Wallis test, indicating no significant difference (*P* < 0.72; 1 × 10^6^ permutation samples). Data are mean ± SD The donors included in **(A)** are indicated in this figure. **(C)** Maximum doublings as a function of percentage of cells that were PD-1+. Data are presented as means, and when included, error bars indicate standard deviations over technical replicates for that donor. The symbols in **(C)** correspond to Rai stage indicated in **(B)**.

As a measure of cell suitability for production, we compared *ex vivo* expansion of T cells from individuals being treated for CLL to those from healthy counterparts. Mixed CD4^+^/CD8^+^ populations of T cells were activated using Dynabeads (α-CD3 + α-CD28) then expanded in media supplemented with serum but without additional cytokines. Cells from healthy donors entered a phase of rapid growth, after which expansion decreased and cells came to rest (Figure 1A). Cells from CLL patients was often less robust, manifested as a shorter period of rapid growth and/or slower rate of doubling; three examples illustrating strong (similar to healthy donors), moderate, and minimal growth are shown in Figure 1A. Toward a systematic understanding of this variability, we examined a larger set of donors (Supplementary Table 1) seeking to identify parameters that can be associated with different degrees of expansion. This report uses the maximum number of doublings reached during growth, illustrated in each profile of Figure 1A by an open symbol, as an indicative measure of proliferative potential during expansion. We first examined Rai stage, a clinical designation based on disease progression (Apelgren et al., 2006). Cells from healthy donors exhibited 5.5 ± 0.4 (mean ± SD, *n* = 5) doublings. Cells from CLL patients showed a wider range, with no dependence on Rai stage (*P* < 0.72, permutation on Kruskal-Wallis test). We next considered the percentage of cells in the starting population expressing the checkpoint inhibitor PD-1 (Arasanz et al., 2017). An overall negative correlation was observed between maximum doublings and PD-1 expression (Figure 1C), but with a dip in doublings for intermediate values of PD-1 expression. Analysis of maximum doublings as a function of sex and IgVH mutation status showed no significant effect of the individual parameters (*P* < 0.43 and *P* < 0.29, respectively, two-tailed *t*-test). Recognizing that cellular functions are central to disease progression, we next turned to more complex measures of cell state.

### Cell Sensitivity to Micropatterned, Activating Signals Is Dependent on PD-1 Expression

**FIGURE 2:**
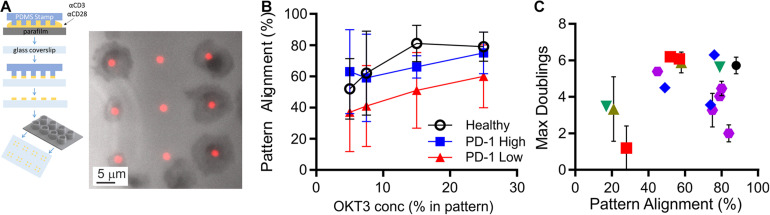
Measurement of cell function from limited samples. **(A)** Microcontact printing was used to pattern isolated features containing activating antibodies to CD3 and CD28, allowing microscopy-based analysis of cell function. These micropatterned surfaces were attached under custom-made, open-bottomed conical chambers, which provide a 20-fold improvement in cell utilization. Cell-substrate contact areas were determined by interference reflection microscopy (gray), which allowed determination of alignment with activating features of α-CD3 + α-CD28 (red). **(B)** Alignment of T cells to the activating features was dependent on both the concentration of α-CD3 antibody (OKT3) and PD-1 expression. Data are mean ± SD from 3 to 14 donors for each condition. An OKT3 concentration of 15% was selected as a standard condition for subsequent experiments. **(C)** Maximum doublings as a function of Pattern Alignment. Data are means, and when included error bars indicate standard deviations over technical replicates for that donor. The symbols in **(C)** correspond to Rai stage as indicated in Figure 1B.

CLL impacts cellular-level functions of T cells, including motility, migration, and activation (Ramsay et al., 2013; Dupre et al., 2015). In this section, we seek to characterize such functions under well-defined conditions, potentially leading to a new quantifier that can be used to determine cell state. These assays typically require observation of live cells, and have been complicated by both the limited number of cells available from diagnostic samples and large, unobservable dead volumes associated with microscopy systems. To address the microscopy-associated limitation, we introduced the use of conical wells to collect cells into a small region of observation. The chambers are based on 96-well plates, with each well concentrating cells that would settle onto the 5-mm diameter bottom surface to a 1-mm diameter observation area (Figure 2A). By concentrating cells onto the observation area, the number of cells needed for an experiment was reduced by a factor of 20, facilitating experiments with smaller diagnostic samples and/or testing of more parameters from a single sample. Here, these chambers were used in conjunction with a second experimental system, protein-micropatterned surfaces for measuring response of living cells (Figure 2A). Microcontact printing (Mayya et al., 1950, 2018; Chen et al., 1997; Shen et al., 2008; Bashour et al., 2014; Kumari et al., 2015) was used to create arrays of 2-μm diameter, circular features containing antibodies to CD3 and CD28 which provide activation and costimulatory signals, respectively. The intervening regions were coated with ICAM-1. This approach was used previously (Shen et al., 2008) to investigate sensitivity of T cells to localized CD3 activation, assayed by measuring the percentage of cells that stopped on and aligned with the features as a function of α-CD3 concentration. Repeating that approach here, primary human T cells from healthy donors aligned with micropatterned features of OKT3 (α-CD3) and 9.3 (α-CD28) as shown in Figure 2A. The amount of CD3 activating signal was controlled by specifying the concentration of OKT3 in the printing solution, as detailed in section “Materials and Methods.” The percentage of cells that aligned with the patterns increased as OKT3 concentration increased. Cells from CLL donors similarly showed increasing alignment with higher concentrations of α-CD3, but also exhibited a dependency on PD-1 expression (Figure 2B). For this analysis, cells with PD-1 expression levels within the 95% confidence interval of healthy donors were designated as “PD-1 low,” while those above this confidence interval were notated as “PD-1 high.” At each OKT3 concentration, cells from the “low” group showed lower alignment with features than the corresponding cells from healthy donors. Surprisingly, this deficit in cell response was lost for cells from the “high” PD-1 group, illustrating the complex relationship between maximum doublings and PD-1 expression suggested in Figure 1C. Notably, these experiments were made practical by the improvement in cell utilization provided by the conical chamber system. Subsequent experiments, facing similar limitations in cell availability, were carried out at an OKT3 concentration of 15% (see section “Materials and Methods”), corresponding to the greatest difference between cells of the healthy and PD-1 low donors. Cell proliferative potential is plotted as a function of pattern alignment at this standardized concentration of 15% OKT3 in Figure 2C. While lower levels of alignment were associated with decreased proliferative potential, the number of doublings reached by cells exhibiting higher alignment varied across the range of observed values; the distribution of maximum doublings for alignment above 60% was not statistically different than those below this cutoff (*P* < 0.61, permutation of Kruskal-Wallis test, 1 × 10^6^ random permutations). Finally, IL-2 secretion by cells adherent to these micropatterned surfaces was measured using a previously described surface capture method (Shen et al., 2008; Bashour et al., 2014). Like pattern alignment and other biomarkers, no clear correlation between maximum doublings and IL-2 secretion alone was observed. Given these results, we next pursued a multi-factor approach toward characterizing cell proliferative potential.

### Clustering Analysis Reveals Three Groups of Donors

**FIGURE 3:**
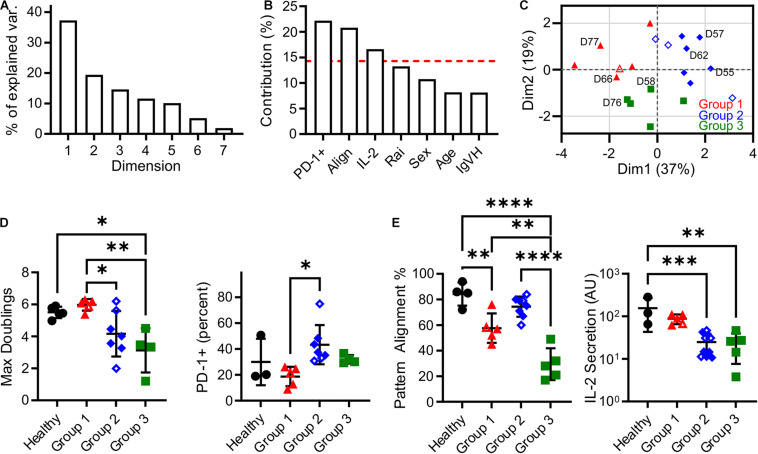
Clustering analysis of cells from CLL patients revealed three Groups that describe proliferative potential. **(A)** Scree plot indicating the percentage of explained variance associated with each Dimension of a seven-factor FAMD analysis. Subsequent analysis focused on Dim1 + Dim2, which explains over 50% of variance. **(B)** Contribution of each factor to Dim1 + Dim2. The red line indicates 14.3%, a threshold representing equal contribution by each factor. **(C)** Analysis by k-medoids clustering using factors with contributions above the threshold indicated in **(B)** produced three Groups, which are coded in this FAMD plot showing Dim1 and Dim2. The labeled donors are examined in more detail in Figure 4. **(D)** Maximum doublings varied as a function of Group assignment. **(E)** PD-1, Alignment, and IL-2 secretion as a function of Group assignment. In all panels, data are mean ± SD ^∗^*P* < 0.05, ^∗∗^*P* < 0.005, ^∗∗∗^*P* < 0.0005, ^*⁣*⁣**^P < 0.0001, using ANOVA and Tukey tests. All comparisons that were significant at α = 0.05 are indicated in this figure. Open symbols represent conditions for which missing data was imputed.

In this section, an unsupervised clustering approach was used to identify patterns in biomarker expression within the populations of T cells isolated from CLL donors. Factors for this analysis included pattern alignment, IL-2 secretion, Rai stage, PD-1 expression, age at time of diagnosis, sex, and IgVH mutation status. Before clustering, Factor Analysis of Mixed Data (FAMD, Figure 3A) was used to identify which factors have the largest impact of explaining data variance. Dimensions 1 and 2 together comprised over 50% of data variability (37.3 and 19.4%, respectively, Figure 3A). As such, we examined the contributions of the seven input factors to combined Dim1 + Dim2. Pattern alignment, IL-2 secretion, and PD-1 expression each contributed over 14.3%, a cutoff representing equal contributions from all factors (Figure 3B), and were thus identified as the factors to be used in k-medoids clustering analysis. A cluster number of three was selected using the elbow method (Supplementary Figure 1), leading to group assignments shown in Figure 3C. Most strikingly, the groups stratify maximum doublings (Figure 3D): Group 2 is significantly lower than Group 1 (*P* < 0.05), while Group 3 is lower than both Healthy and Group 1 cells (*P* < 0.05 and *P* < 0.005, respectively). These assignments thus provide a single parameter that describes cell expansion potential without the complex relationships observed for individual factors (Figures 1B,C, 2C). These group assignments also provided insight into the three factors that were used in clustering—PD-1, pattern alignment, and IL-2 secretion (Figure 3E). Intriguingly, clustering provided more distinct stratification of pattern alignment than max doublings (four comparisons that were significant at α = 0.05, compared to three), but alignment showed a different order of response with Group 2 being higher than the others. A similarly altered order was observed for PD-1 expression. Finally, IL-2 secretion showed an ordering that was similar to max doublings, suggesting a connection between doublings and cytokine secretion, but fewer comparisons were significant at α = 0.05.

It is noted that the clustering and data imputation algorithms used here incorporate randomization. Consequently, the stability of these analyses was tested through two types of resampling. The first is bootstrapping, in which 500 data sets were generated by random selection with replacement and then analyzed using the methods applied to the original data set. The frequency at which each donor was assigned to a given Group is listed in Supplementary Table 2, showing that the groups reported in our full data set (Supplementary Table 1) are stable; only one donor (D59) was assigned to a group different from the bootstrapped data. Data were then analyzed by subsampling, in which 500 data sets representing 90% of the original were generated by random sampling without replacement. As shown in Supplementary Table 1, these assignments followed the original analysis, indicating that those conclusions are not sensitive to the number of individual donors. Finally, bootstrapping was conducted on percentage of variance explained by Dim1 + Dim2 in the FAMD analysis. Analysis of 500 bootstrap sets determined a 95% confidence interval of 53.3–71.0%, placing it above the 50% criteria.

### Cell Motility Varies Between Groups and PD-1 Expression

**FIGURE 4:**
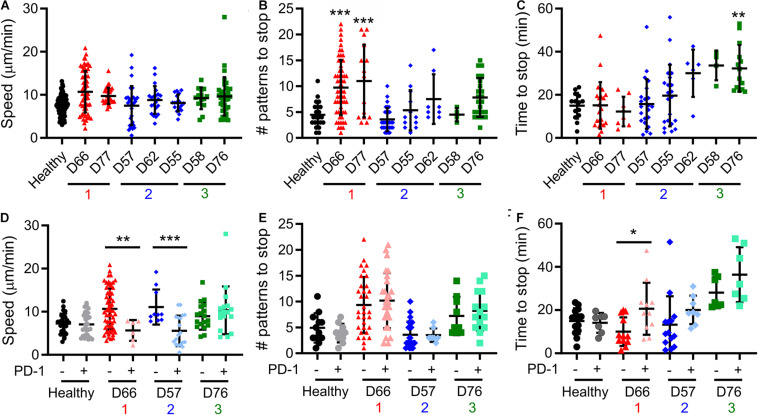
Cell motility varies between Groups. **(A–C)** Live-cell microscopy over the first 60 min of cell-substrate interaction reveal different behaviors in motility. Cell centroid position was tracked and analyzed for average speed **(A)**, the number of patterns that a cell crossed before coming to a halt **(B)**, and time until such a halt **(C)**. Donors included in this analysis are labeled in the FAMD plots of Figure 3. **(D–F)** Prelabeling of cells with an α-PD-1 antibody allowed separate analysis of PD-1+ and PD-1− cells in the same experiment. In all panels, each symbol indicates an individual trajectory. The red, blue, and green numbers below the x axes indicate the Group assignments established in Figure 3. Data are mean ± SD of all cells tracked in 1–2 independent experiments. *P < 0.05, **P < 0.005, ***P < 0.0005 compared in **(A–C)** to healthy donors and in **(D–F)** between PD-1 positive and negative cells for that donor. Additional comparisons are detailed in the main text.

A notable result presented above is that pattern alignment is a major contributor to Dim1 + Dim2 (Figure 3B), and is also stratified by the cluster assignments (Figure 3E). To understand the cellular processes underlying pattern alignment, we examined the motion of cells following contact with a micropatterned surface (Supplementary Movie 1), collecting three complementary measures of cell motion from these trajectories. The first was motility speed, which reflects exploration of the ICAM-1-presenting surfaces. No significant variation in speed was observed across CLL and healthy donors (Figure 4A). The next two measures focused on cells as they encountered and came to a stop (defined as a halt in long-range movement for at least 3 min) on activating features of α-CD3 + α-CD28; these cells represent the ones that aligned with the pattern. The number of features a cell encountered before stopping provides insight into the sensitivity of cells to activation. Cells from Group 1 moved over more features than cells from Group 2, Group 3, and also healthy donors (Figure 4B) suggesting lower sensitivity to activation. As a complementary readout, the time from the beginning of the trajectory to stopping on an α-CD3 + α-CD28 feature was also measured. Cells in Group 3 showed the longest trajectory duration (Figure 4C). These results collectively suggest that proliferative potential is associated with different patterns of cell motility and sensitivity to activation. Specifically, longer periods of motion before coming to a stop are associated with lower maximum doublings, as illustrated for D76. However, this relationship is complex, since Group 1 showed lower sensitivity to activation with regards to the number of features crossed before stopping. Finally, cell motility was compared as a function of PD-1 expression by labeling cells for PD-1 prior to use in migration assays. Separating cells in this manner revealed that PD-1− cells from D66 (Group 1) moved faster than their PD-1 + counterparts (Figure 4E), and also cells from healthy donors, regardless of PD-1 expression (*P* < 0.005). The number of features experienced before stopping for cells from D66 was greater than for healthy donors, regardless of PD-1 expression (*P* < 0.05), in keeping with Figure 4B. These differences are further reflected in a longer time to stop for PD-1+ cells from D66 compared to their PD-1− counterparts (Figure 4F). A similar increase in migration speed for PD-1− cells vs. PD-1+ counterparts was observed for D57 (Group 2), but these differences were not significant compared to healthy donors. No effect of PD-1 expression on migration was observed for D76 (Group 3).

## Discussion

Cancer, like many afflictions, is multifaceted and diverse requiring specification of treatment course around the disease state and individual. This extends into surprising facets of the tools used for therapy. For example, we recently demonstrated that replacing the mechanically stiff plastic beads that are routinely used to activate T cells with a softer material can enhance subsequent expansion, providing more cells from an initial starting population and rescuing production of cells from individuals with CLL (Dang et al., 2018). Intriguingly, the stiffness of the material that produced optimal growth of cells varied between CLL donors. Through this study, we seek a framework for describing and understanding the differences in proliferative potential observed between CLL patients.

Initial attempts to use single factors such as Rai stage (as T cell expansion capabilities decrease with disease progression; Bonyhadi et al., 2005) and PD-1 expression (which is elevated in exhausted T cells; McLane et al., 2019) to capture variability in cell proliferation had modest success (Figures 1B,C). As such, we expanded the set of parameters to include measures of cell function, specifically cytokine secretion and the ability to align with micropatterned features on an activated surface. Individually, these measures provided limited new insight. We subsequently turned to multi-factor machine learning approaches, which have had success in classification of various tumor models (Gorris et al., 1950; Zucchetto et al., 2011; Chen and Mellman, 2017; Gonnord et al., 2019). Unsupervised clustering based on PD-1, alignment, and IL-2 provided a compelling approach for categorizing cells from CLL patients into three groups, which differed with respect to proliferative potential, an independent factor that was not included in the analysis but is important to cell production. Designing future studies around this clustering approach may provide a streamlined method for understanding cell exhaustion and developing tools for improving cell expansion.

Pattern alignment emerged as a key factor describing T cell response. In FAMD analysis, alignment contributed to Dim1 + Dim2 to an extent almost equal to PD-1 expression (Figure 3B). Moreover, of the six potential pairwise comparisons possible between Groups and Healthy donors, four of these were statistically significant for pattern alignment. By comparison, PD-1 and IL-2 secretion showed fewer significant comparisons, suggesting that alignment provides the greatest stratification between groups. However, pattern alignment is a complex process, involving adhesion to a micropatterned surface, motion across that surface, interaction with multiple activating features, and finally (in the window of our assay) cessation of motility. Most prominently, cells from Group 1 passed over more features before stopping than the other groups and healthy donors (Figure 4B). Compared to uniformly coated surfaces, these micropatterned features more accurately capture the physiological process of T cells encountering and even competing for a limited number of conjugate cells (Mayya et al., 1950, 2018). As described in the Results section, a simple interpretation of this is that passing over multiple patterns reflects the sensitivity of cells to activation, or the need to integrate multiple encounters before cessation of motion, which is associated with TCR-induced actin polymerization, through proteins such as Wiskott-Aldrich syndrome protein (WASP), overcoming polarization of cytoskeletal dynamics and tension (Kumari et al., 2020). However, another interpretation is that moving over multiple features can reflect persistence of cell motion, with a stop being more likely to happen at the same phase of motion as a change of direction. Maiuri et al. (2012) elegantly demonstrated that persistence and cell speed are correlated, developing a model in which actin flow maintains polarization (Maiuri et al., 2015). Correspondingly, the increase in features passed over by cells in Group 1 is associated with faster motion, but only for PD-1− cells (D66, Figure 4D). PD-1 expression, even in the absence of ligand on the underlying surface, reduced cell speed while not affecting the number of features passed over, suggesting a further complexity in how processes are balanced in cell migration. Intriguingly, Group 1 showed lower pattern alignment than healthy donors (potentially reflecting increased motion persistence) but strong proliferative potential. Perhaps counterintuitively, it is possible that modulating cell alignment by increasing migration speed could lead to improved cell activation and production for immunotherapy. A clearer understanding of how cytoskeletal polarization and dynamics interact is needed to more fully realize this potential.

## Data Availability Statement

The original contributions presented in the study are included in the article/Supplementary Material, further inquiries can be directed to the corresponding author/s.

## Ethics Statement

The studies involving human participants were reviewed and approved by the Dana-Farber Cancer Institute Columbia University. The patients/participants provided their written informed consent to participate in this study.

## Author Contributions

JL and LK designed the study. JL, SS, MK, and LK performed data collection and analysis. SF and JB provided expertise on CLL and management of donor samples. All authors contributed to the article and approved the submitted version.

## Conflict of Interest

The authors declare that the research was conducted in the absence of any commercial or financial relationships that could be construed as a potential conflict of interest.

## Supplementary Material

Supplementary Figure 1 Determination of cluster number.

Supplementary Table 1 Cells from healthy (H3–H9) and CLL (D2–D78) donors.

Supplementary Table 2 Analysis of clustering stability.

Supplementary Movie 1 Motility of T cells on micropatterned surfaces.
