# Supplementary material for: Multi-Factor Clustering Incorporating Cell Motility Predicts T Cell Expansion Potential
Source: Front Cell Dev Biol. 2021 Apr 9;9:648925. doi: 10.3389/fcell.2021.648925 (PMC8063612; doi:10.3389/fcell.2021.648925)
Supplement: Supplementary Figure 1 — Determination of cluster number. [file Data_Sheet_1.PDF]

Supplementary Material

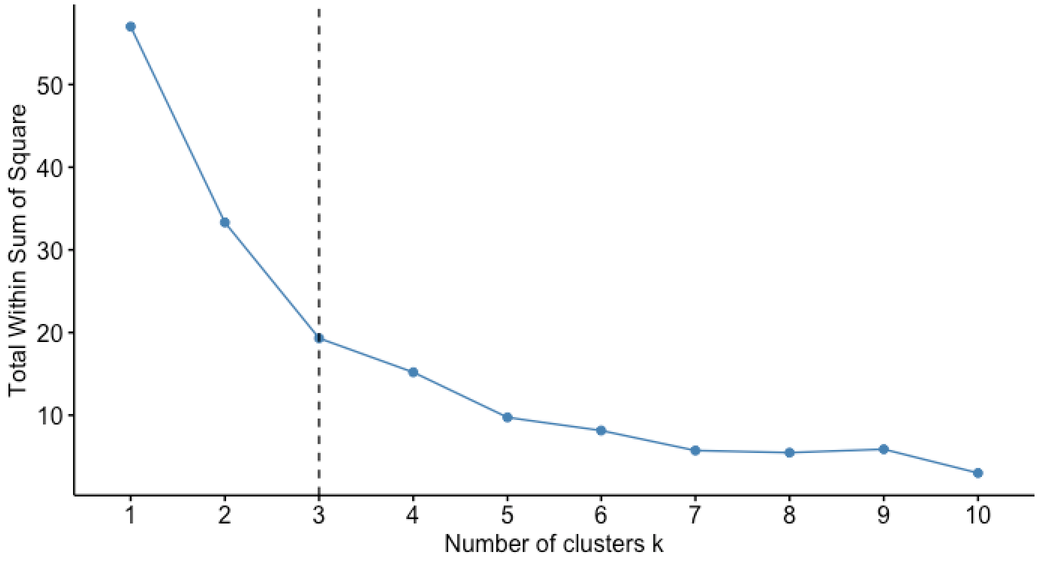

Supplementary Figure S1. Determination of cluster number. Plot of sum of squared error as a function of k-medoid cluster number. Elbow analysis was used to identify an optimal cluster number of 3.

Patient No	Age	IgVH	PD-1 (%)	Rai	Sex	Pattern Alignment (%)	IL-2	Max Doublings	Group
D2	59	MUT	35.20	4	M	75	10.75	3.28	2
D44	81	UNMUT*	74.88	1	M	75*	11.26*	6.20	2
D46	71	MUT*	33.04	4	F	84	30.79	2.00	2
D47	71	MUT	56.59	4	F	60	11.26		2
D48	56	UNMUT	45.59	1	M	71	14.62		2
D54	63	UNMUT	26.03	4	F	45	110.09	5.39	1
D55	88	UNMUT	48.36	3	M	74	42.61	3.56	2
D57	84	MUT	37.38	4	M	80	14.79	4.46	2
D58	52	UNMUT	36.56	3	F	49	46.33	4.50	3
D59	45	MUT	8.29	3	F	13	110.81*		1
D62	42	UNMUT	43.50	4	M	79	36.74	4.02	2
D65	69	UNMUT	39.67	2	M	32	3.79		3
D66	52	MUT	24.70	0	F	57	62.33	6.09	1
D68	83	MUT	12.85	3	M	76	90.53	6.30	1
D69	48	MUT*	30.87	2	M	79	46.33*	5.63	2
D74	59	MUT*	21.19	1	F	58	66.98	5.88	1
D75	76	UNMUT	32.09	0	F	28	27.11	1.20	3
D76	57	MUT	29.35	2	F	17	14.48	3.48	3
D77	47	MUT	8.89	0	M	52	110.81	6.20	1
D78	69	MUT	30.07	1	F	21	25.91	3.34	3
H3								5.45	
H4	34		19.00		F	94	283.4	4.98	
H6	49		20.18		F	85	119.69	5.76	
H8								5.42	
H9	49		50.70		M	86	65.63	5.91	

Supplementary Table S1. Cells from healthy (H3 – H9) and CLL (D2 – D78) donors were analyzed on the basis of factors arising from clinical presentation (Age at diagnosis, IgVH mutation, percent PD-1 positive expression, Rai stage, and Sex) and outputs of cell function (Pattern Alignment, IL-2 secretion, and Maximum Doublings). Analysis by k-medoids clustering identified three Groups, indicated in the right-most column. * indicates imputed data.

Patient No	Assigned Group		Bootstrap Fraction of assignments					90% Subsample Fraction of assignments			
			Group Max	1	2	3		Group Max	1	2	3
D2	2		2	0.03	0.97	0.01		2	0.00	1.00	0.00
D44	2		2	0.04	0.87	0.09		2	0.00	0.98	0.02
D46	2		2	0.06	0.94	0.01		2	0.00	1.00	0.00
D47	2		2	0.03	0.91	0.06		2	0.00	1.00	0.00
D48	2		2	0.01	0.97	0.02		2	0.00	1.00	0.00
D54	1		1	0.95	0.01	0.04		1	1.00	0.00	0.00
D55	2		2	0.05	0.94	0.01		2	0.00	1.00	0.00
D57	2		2	0.03	0.97	0.00		2	0.00	1.00	0.00
D58	3		3	0.38	0.20	0.42		3	0.34	0.03	0.64
D59	1		3	0.40	0.01	0.59		1	0.52	0.00	0.48
D62	2		2	0.05	0.94	0.01		2	0.00	1.00	0.00
D65	3		3	0.04	0.04	0.91		3	0.00	0.00	1.00
D66	1		1	0.84	0.06	0.10		1	0.94	0.00	0.06
D68	1		1	0.94	0.01	0.05		1	1.00	0.00	0.00
D69	2		2	0.32	0.67	0.01		2	0.31	0.69	0.00
D74	1		1	0.92	0.02	0.06		1	0.99	0.00	0.01
D75	3		3	0.05	0.01	0.93		3	0.00	0.00	1.00
D76	3		3	0.04	0.01	0.95		3	0.00	0.00	1.00
D77	1		1	0.94	0.01	0.05		1	1.00	0.00	0.00
D78	3		3	0.06	0.01	0.94		3	0.00	0.00	1.00

Supplementary Table S2. Analysis of clustering stability. The data set presented in Table S1 was reanalyzed using bootstraping (sampling with replacement) and subsampling (random selection of 90% of samples, without replacement) methods, n = 500 data sets for each analysis. Data marked as imputed in Table S1 was reimputed for each resampled data set. Each data set was then analyzed by k-medoids clustering. The fraction of outcomes resulting in assignment to each indicated group is listed in this table. The group with the highest fraction of assignments is listed in the Group Max column.

Supplementary Movie S1. Motility of T cells on micropatterned surfaces. Surfaces containing micropatterned features of α-CD3 + α-CD28 (red) were produced by microcontact printing. Migration of primary human T cells (green, brightfield) was tracked over a 60-minute period. Blue arrows illustrate cells that have stopped migrating.
